# *Helicobacter pylori* Genotypes Associated with Gastric Histo-Pathological Damages in a Moroccan Population

**DOI:** 10.1371/journal.pone.0082646

**Published:** 2013-12-09

**Authors:** Samia Alaoui Boukhris, Afaf Amarti, Karima El Rhazi, Mounia El Khadir, Dafr-Allah Benajah, Sidi Adil Ibrahimi, Chakib Nejjari, Mustapha Mahmoud, Abdellah Souleimani, Bahia Bennani

**Affiliations:** 1 Laboratoire de Microbiologie et Biologie Moléculaire, Equipe micro-organismes et facteurs oncogènes, Faculté de médecine et de Pharmacie de Fès (FMPF), Université Sidi Mohammed Ben Abdellah (USMBA), Fès, Maroc; 2 Service d’Anatomie pathologique CHU Hassan II, Fès, Maroc; 3 Laboratoire d’Epidémiologie et de recherche clinique, FMPF, USMBA, Fès, Maroc; 4 Service d’Hépato gastro-entérologie CHU Hassan II de Fès, Equipe Maladies de l’appareil digestif (FMPF), Fès, Maroc; 5 Service de Bactériologie CHU Hassan II, Fès, Maroc; 6 Laboratoire de Biotechnologie, Faculté des sciences Dhar el Mehraz, USMBA, Fès, Maroc; 7 Laboratoire de Biologie des cancers, FMPF, USMBA, Fès, Maroc; Veterans Affairs Medical Center (111D), United States of America

## Abstract

*H. pylori* persistent infection induces chronic gastritis and is associated with peptic ulcer disease and gastric carcinoma development. The severity of these diseases is related to human’s genetic diversity, *H. pylori* genetic variability and environmental factors. To identify the prevalence of histo-pathological damages caused by *H. pylori* infection in Moroccan population, and to determine their association to *H. pylori* genotypes, a prospective study has been conducted during 3 years on patients attending the gastroenterology department of Hassan II University Hospital (CHU) of Fez, Morocco. A total of 801 Moroccan adults’ patients were recruited; *H. pylori* was diagnosed and genotyped by PCR in biopsy specimens and histological exam was performed. We found a high rate of glandular atrophy. Chronic inflammation, neutrophil activity and glandular atrophy showed statistically significant association with *H. pylori* infection. However, intestinal metaplasia was inversely associated to this infection and no association was observed with gastric cancer cases. A statistically significant association was found between intestinal metaplasia and *vacAs1* and *vac Am1* genotypes in patients aged 50 years and more but not in younger. This last genotype is also associated to gastric cancer. In this study, gastric cancer showed no significant association with *H. pylori*. Further studies are warranted to determine the role of other etiological agents such as Epstein-Barr virus, human papillomavirus and possibly environmental and dietetic factors in the occurrence of this pathology.

## Introduction

*Helicobacter pylori* (*H. pylori*) infection affects almost half of the world’s population. The persistent infection induces chronic inflammation of the gastric mucosa and peptic ulcers. *H. pylori* is also a major risk factor for gastric cancer [1,2]. The severity of the disease is related to human genetic diversity, environmental factors and *H. pylori* genetic variability [3]. *VacA* gene encodes a vacuolating cytotoxin which is excreted by *H. pylori* and leads to epithelial cells damages. This gene is present in all strains, and comprises variable regions (s, m and i). The s region (encoding the signal peptide) exists as s1 or s2 allele. The m region (middle) occurs as m1 or m2 allele. The mosaic combination of s and m allelic types determines the level of cytotoxin produced which has been associated to pathogenicity of the bacterium [4]. The *cagA* gene (cytotoxin-associated gene) is considered a marker of the pathogenicity island gene (*cag PAI*) presence. Infection with *H. pylori cagA* ^*+*^ strains has been associated with an increased risk of atrophic gastritis and gastric cancer development [4]. In Morocco, there is no published data regarding the occurrence of histo-pathological damages and their correlation with *H. pylori* genotypes. The aim of this study is to determine the prevalence of different histopathological lesions, especially the premalignant ones, in infected patients and their correlation to *H. pylori* infection and also with *vacA* genotypes and *cagA* status.

## Materials and Methods

### Sample collection

Between May 2009 and January 2013, we recruited all the patients aged 15 years and more, attending the Gastroenterology Department of Hassan II University Hospital of Fez and undergoing endoscopy for diagnosis of abdominal pain or discomfort. All patients aged below 15 years or who were on medications (antibiotics, proton pump inhibitors) for the last 3 months, as well as pregnant or nursing women were excluded from this study. Consenting patients had a personal interview before endoscopy. Parental consent was obtained on the behalf of the participants under the age of 18. In the case of illiterate or semi-literate patients, the written consent was read to them by the interviewer.

In each participant, six biopsies samples were taken from the antrum and the middle body and used for molecular diagnosis of *H. pylori* and also for histo-pathological exam. Four gastric biopsies (2 fundic and 2 antral) from each subject were fixed in buffered formalin (10%) and stained with hematoxyline-eosin and Giemsa. Histopathological evaluation of samples from both settings was performed according to the Modified Sydney system [5] by at least two experimented pathologists. Gastric cancer cases have been classified as adenocarcinoma, signet ring cell carcinoma (SRCC), undifferentiated carcinoma and Malt lymphoma. Presence or absence of *H. pylori* on gastric biopsies was performed on histological slides and scored in a semi quantitative approach from 1+ to 3+ depending on the amount of germs in glandular crypts.

### *H.pylori* molecular diagnosis and genotyping

*H. pylori* DNA extraction was done on two gastric antral biopsies as previously described [6]. Diagnosis of *H. pylori* was done by PCR using *glmM* primers [7]; all positives specimens were subjected to PCR using genotype specific primers. *cag A* status and *vacA* genotypes were determined by multiplex PCR using primers and conditions previously described to amplify *cag A* and the signal (s) and middle (m) regions of *vac A* [8,9]. PCR using a second primer pair was done to determine *cag A* status as previously described [10] and *cag PAI* empty site PCR was used to confirm this status [11]. Separately, both positive and negative controls were included. The PCR products were resolved in 2% agarose gel, stained with ethidium bromide and visualized under an UV source. The strains were considered *cagA* positive when at least one of the *cagA* reactions was positive and *cagA PAI* PCR was negative.

### Statistical analysis

Statistical analysis was performed using SPSS software (Statistical Product and Services Solutions, version 17, SPSS Inc, Chicago, Il, USA) to analyze data. In univariate analysis, potential explicative factor for gastric damages was determined; and association between gastric lesions and genotypes was tested independently in two groups, defined according to age group (patients aged less than 50 years were grouped in age group1 and those within 50 years and older in age group 2). All Chi^2^ test results with P-values less than 0.05 were considered statistically significant.

This prospective study was approved by the Ethical Committee of the University Hospital of Fez.

## Results

### Studied population

A total of 801Moroccan adult patients were recruited in this prospective study (424 men [52.93%], and 337 women [47.07%]), aged between 15 and 99 years with mean age of 49.2 years (Standard Deviation [SD] 16.3 years). The percentage of patients with persistent exposure to smoking (active or passive) was 26.10% (202/774) whilst an 8.9% reported consumption of alcohol. Approximately two-thirds of recruited patients (n=509/780; 65.3%) were from urban areas. 

Molecular diagnosis showed that 63.13% of women were *H. pylori* infected versus 56.6% of men (p=0.06). This infection was significantly higher in patients aged 50 years and less (66.93%) than in older ones (53.19%) (p=0.00007). No association was found between *H. pylori* infection and area, tobacco smoking or alcohol consumption (Table 1). 

**Table 1:** *H. pylori* status associated to risk factors.

		***H. pylori***		**p value**	**Total**
		Positive n (%)	Negative n (%)		n (%)
**Gender**	Men	240/424 (56.6)	184/424 (43.4)	0.06	424/801 (52.9)
	Women	238/377 (63.1)	139/377 (36.9)		377/801 (47.1)
**Age**	<50	253/378 (66.9)	125/378 (33.1)	0.00007	378/801 (47.2)
	>50	225/423 (53.2)	198/423 (46.8)		423/801 (52.8)
**Area**	Urban	309/509 (60.7)	200/509 (39.3)	0.5	509/780 (65.3)
	Rural	158/271 (58.3)	113/271 (41.7)		271/780 (34.7)
**Tobacco smoking**	Yes	130/204 (63.7)	74/204 (36.3)	0.19	204/780 (26.2)
	No	337/576 (58.5)	239/576 (41.5)		576/780 (73.8)
**Alcohol**	Yes	37/69 (53.6)	32/69 (46.4)	0.26	69/780 (8.8)
	No	430/711(60.5)	281/711(39.5)		711/780 (91.2)
**Total**		478/801 (59.7)	323/801 (40.3)		

*CagA* status and *vacA* s and m regions genotypes were determined for all positive specimens. As reported in table 2, 59.6% of cases were *cagA* positives and *vacA* single allele (s or m) (31.27%) is the most predominant profile followed by *vacA* s2m2 (29.28%) subtype. The *vacA* s2m1 subtype has been observed in 4 cases. When considering each genotype separately, s2 and m2 were the most predominant genotypes and were detected in 51.74% and 64.23% cases, respectively.

**Table 2:** *H. pylori* genotypes description.

**Genotype**	**Subtype**	**n (%)**
***Vac A***	s1m1	81/478(16.9)
	s1m2	33/478(6.9)
	s2m1	4/478(0.8)
	s2m2	118/478(24.7)
	Single s/m	126/478 (26.4)
	Multiple	41/478(8.6)
	Not genotyped	75/478(15.7)
	s1	166/344 (48.3)
	s2	178/344 (51.7)
	m1	98/274 (35.8)
	m2	176/274 (64.2)
***Cag A***	Positive	248/416(59.6)
	Negative	168/416(40.4)

The histological exam carried out on 791 specimens, showed a high prevalence of chronic inflammation (CI) (91.51%, n=724/791), glandular atrophy (GA) (81.42%, n=644/791) and neutrophil activity (NA) (73.9%, n=585/791). Lesions known as pre-neoplastic such intestinal metaplasia (IM) and dysplasia were detected in 14.66% (116/791) and 1.52% (12/791) cases respectively. However, gastric cancer (GC) was diagnosed in 6.19% (49/ 791) cases with predominance of a signet ring cell carcinoma (SRCC) (41.67%, n=20/48), followed by adenocarcinoma (37.5%, n=18/48)) and finally by MALT lymphoma (20.38%, n=10/48). *H. pylori* was detected in 63.7% cases using histopathology. 

### Gastric lesions and associated risk factors

Tissue damages were correlated with age, gender, area, tobacco smoking, alcohol consumption and *H. pylori* infection. The results showed that all lesions types were predominant in patients aged more than 50 years old with significant association in GA, IM and GC cases (p<0.05). Statistically significant association was also found between gender and IM with predominance in men (18.9% vs 9.9% in women) (p=0.0002). The same tendency was observed in gastric cancer, but the association did not reach statistical significance (Table 3).

**Table 3:** Gastric damages and risk factors.

		**CI**	**NA**	**GA**	**IM**	**GC**	**Total**
**Age**	<50 y	340/372(91.4)	278/372(74.7)	293/372(78.8)	38/372(10.2)	17/372(4.6)	378/801(47.2)
	>50 y	384/419(91.6)	307/419(73.3)	351/419(83.8)	78/419(18.6)	32/419(7.6)	423/801(52.8)
	p value	0.5	0.34	0.04	0.0005	0.04	
**Gender**	Male	389/418(93.1)	310/418(74.2)	339/418(81.1)	79/418(18.9)	30/418(7.2)	424/801(52.9)
	Female	335/373(89.8)	275/373(73.7)	305/373(81.8)	37/373(9.9)	19/373(5.1)	377/801(47.1)
	p value	0.06	0.47	0.44	0.0002	0.14	
**Smoking**	*Yes*	189/200(94.5)	159/200(79.5)	157/200(78.5)	31/200(15.5)	16/200(8)	204/780(26.2)
	*No*	514/570(90.2)	411/570(72.1)	471/570(82.6)	81/570(14.2)	32/570(5.6)	576/780(73.8)
	p value	0.03	0.02	0.1	0.36	0.15	
**Alcohol**	*Yes*	64/68(94.1)	49/68(72.1)	52/68(76.5)	12/68(17.6)	5/68(7.4)	69/780(8.8)
	*No*	639/702(91)	521/702(74.2)	576/702(82.1)	100/702(14.2)	43/702(6.1)	711/780(91.2)
	p value	0.27	0.39	0.16	0.27	0.42	
**Living area**	Rural	247/265(93.2)	198/265(74.7)	230/265(86.8)	47/265(17.7)	22/265(8.3)	271/780(34.7)
	Urban	456/505(90.3)	372/505(73.7)	398/505(78.8)	65/505(12.9)	26/505(5.1)	590/780(65.3)
	p value	0.10	0.41	0.003	0.04	0.06	
H.pylori*	Positive	440/473 (93)	375/473(79.3)	399/473(84.4)	50 /473(10.6)	25/473(5.3)	478/801(59.7)
	Negative	284/318(89.3)	210/318 (66)	245 /318(77)	66 /318(20.8)	24/318(7.5)	323/801(40.3)
	p value	0.04	0.00002	0.006	0.00006	0.12	

^*^ *H.pylori* PCR result

Tobacco smoking was statistically significantly associated with CI and NA. However, no association was observed between alcohol consumption and histological damages. Histological pattern was associated with the area of residence. Patients from rural areas had a higher rate of GA (p=0.003) and/or IM (p=0.04) compared to those from urban areas. The same trend was observed in GC cases but the analysis had weak statistical significance (p=0.06) (Table 3). 

On the basis of PCR results, the association of *H. pylori* infection and tissue damages was verified. As reported in Table 1, *H. pylori* was positively associated to CI, NA and GA and negatively associated with IM. *H. pylori* infection was detected most frequently in patients with SRCC than ADK and Malt lymphoma with rates of 60%, 55.6% and 20% respectively. 

### *H. pylori* genotypes and gastric damages

After adjustment for age, we found a statistically significant association between *H. pylori* infection and CI, GA, NA in patients aged less than 50 years. An association was also found between *H pylori* and severe lesions (GA and metaplasia) in individuals aged 50 years or more (Table 4). 

**Table 4:** *H. pylori* Genotypes associated to gastric damages in age groups 1 and 2.

		**<50 ans**			**>50 ans**		
		**Chronic inflammation**			**Chronic inflammation**		
		Yes	No	p	Yes	No	p
**PCR**	Positive	69.1 (235/340)	43.8 (14/32)	0.003	53.4 (205/384)	54.3 (19/35)	0.9
***Cag A***	Positive	65 (132/203)	50 (6/12)	0.29	54.4 (98/180)	58.82 (10/17)	0.72
***VacA*s**	s1	52.3 (92/176)	22.2 (2/9)	0.07	43.2 (60/139)	62.5 (10/16)	0.1
	s2	47.7 (84/176)	77.8 (7/9)		56.8 (79/139)	37.5 (6/16)	
***VacA*m**	m1	40.1 (57/142)	14.3 (1/7)	0.17	30.9 (34/110)	41.7 (5/12)	0.44
	m2	59.9(85/142)	85.7 (6/7)		69.1 (76/110)	58.3(7/12)	
		**Neutrophil activity**			**Neutrophil activity**		
		Yes	No	p	Yes	No	p
**PCR**	Positive	71.6 (199/278)	53.2 (50/94)	0.001	57.3 (176/307)	42.9 (48/112)	0.008
***Cag A***	Positive	68.4 (119/174)	46.3 (19/41)	0.008	55.8 (86/154)	51.2 (22/43)	0.58
***VacA*s**	s1	54.6 (83/152)	33.3 (11/33)	0.02	46 (58/126)	41.4 (12/29)	0.64
	s2	45.4 (69/152)	66.7(22/33)		54(68/126)	58.6 (17/29)	
***VacA*m**	m1	41.2 (54/131)	22.2 (4/18)	0.12	29.2 (28/96)	42.3 (11/26)	0.202
	m2	58.8 (77/131)	77.8 (14/18)		70.8 (68/96)	57.7 (15/26)	
		**Glandular atrophy**			**Glandular atrophy**		
		Yes	No	p	Yes	No	p
**PCR**	Positive	68.6 (201/293)	60.8 (48/79)	0.18	56.4 (198/351)	38.8 (26/68)	0.006
***Cag A***	Positive	63.7 (109/171)	65.9 (29/44)	0.78	53.1 (93/175)	68.2 (15/22)	0 .181
***VacA*s**	s1	49.3 (74/150)	57.1 (20/35)	0.4	44.6 (62/139)	50 (8/16)	0.68
	s2	50.7 (76/150)	42.9 (15/35)		55.4 (77/139)	50 (8/16)	
***VacA*m**	m1	40.7 (50/123)	30.8 (8/26)	0.34	29.3 (32/109)	53.8 (7/13)	0.073
	m2	59.3 (73/123)	69.2 (18/26)		70.6 (77/109)	46.2 (6/13)	
		**Intestinal metaplasia**			**Intestinal metaplasia**		
		Yes	No	p	Yes	No	p
**PCR**	Positive	55.3 (21/38)	68.3 (228/334)	0.1	37.2 (29/78)	57.2 (195/341)	0.001
***Cag A***	Positive	70 (14/20)	63.6 (124/195)	0.56	50 (14/28)	55.6 (94/169)	0.57
***VacA*s**	s1	42.9 (6/14)	51.5 (88/171)	0.53	70 (14/20)	41.5 (56/135)	0.016
	s2	57.1 (8/14)	48.5 (83/171)		30 (6/20)	58.5 (79/135)	
***VacA*m**	m1	46.2 (6/13)	38.2 (52/136)	0.57	53.3 (8/15)	29 (31/107)	0.058
	m2	53.8 (7/13)	61.8 (84/136)		46.7 (7/15)	71 (76/107)	
		**Cancer**			**Cancer**		
		Yes	No	p	Yes	No	p
**PCR**	Positive	64.7 (11/17)	67 (238/355)	0.8	43.8 (14/32)	54.3 (210/387)	0.2
***Cag A***	Positive	54.5 (6/11)	64.7 (132/204)	0.49	83.3 (10/12)	53 (98/185)	0.04
***VacA*s**	s1	50 (4/8)	50.8 (90/177)	0.96	66.67 (8/12)	43.4 (62/143)	0.11
	s2	50 (4/8)	49.2 (87/177)		33.33 (4/12)	56.6 (81/143)	
***VacA*m**	m1	42.86 (3/7)	38.7 (55/142)	0.82	60 (6/10)	29.5 (33/112)	0.04
	m2	57.14 (4/7)	61.3 (87/142)		40 (4/10)	70.5 (79/112)	

Values were expressed % (n).

Most patients with positive *H. pylori* (93%) also had chronic inflammation (p=0.04). When stratified by age, 69.12 % of patients with this lesion were *H. pylori* positives in age group 1 (p=0.003). However, no statistically significant association was observed in the age group 2. *VacA* and *cagA* genotypes showed no association to this histological profile.

In patients with NA, *H. pylori* was detected in 64.1% cases (p=0.00003) with 71.6% cases in age group 1 and 57.3% in age group 2. Those associations were significant with p value of 0.001 and 0.008, respectively. This histological profile showed a significant association with *cagA*+ and also with *vac A s1* genotype in age group 1. However, no association was found between NA and *H. pylori* genotypes in age group 2. In patients with GA, *H. pylori* was detected in 62% cases (p=0.005) with rate of 68.6% in age group 1 and 56.4% in age group 2, the latter being statistically significant (p=0.006). Nevertheless, no association was found between GA and the various *H. pylori* genotypes. 

For severe lesions, *H. pylori* was detected in 43.1% (p= 0.00007) of IM cases and in 51.02% of GC cases (p=0.19). The negative association between *H. pylori* and IM was statistically significant in age group 2 with a rate of 37.2% (29/78) (p=0.001). In this age group and for the same lesion, a statistically significant association was found with *vac A*s1 genotype (vs s2) (p=0.016) and with *vac A* m1 genotype (vs m2) (p=0.058).

In cancer cases, a statistically significant association was detected with *cagA* + (p= 0.04) and *vac A* m1 genotype (vs m2) (p=0.04) in age group 2. In this age group, two third of cancer cases *H.pylori* infected were *vac A*s1 but the association did not reach statistical significant value (p=0.1) (Table 4). When studying association of *H .pylori* genotypes with GC types, *vacA* s1 and m1 subtypes were more predominant in SRCC type with 63.6% and 71.4% respectively, versus 42.9% and 42.9% in adenocarcinoma cases, respectively. However, *H. pylori cagA* positive strains were predominant in both types (63.6% in SRCC and 66.7% in adenocarcinoma cases). 

## Discussion

In this prospective study, 801 gastric biopsies samples were analyzed by histopathology and PCR. This last technique has been described in several studies as more sensitive and convenient reproducible method than histopathology [12]. For this reason, the results of *H. pylori* molecular diagnosis were used to study different associations. The interest of histopathology is the fact that it offers the possibility to detect epithelial gastric damages, to classify them in different categories and to assess their severity. Moreover it is the only way to detect high risk lesions or in some cases the presence of associated invasive carcinoma [12-15].

*H. pylori* induces chronic gastritis in virtually all infected patients. This gastritis leads to more sever gastric pathologies, notably, peptic ulcer disease, atrophic gastritis and gastric cancer [16]. The progress of disease seems to depend on bacterial genotypes, host and environmental factors. Some studies have reported a significant correlation between *H. pylori* genotypes and gastric histological damages [17-19]; whilst others reported a variability of genotypes distribution according to nationalities or geographical area [20]. To our knowledge, this is the first study conducted in North Africa and in Morocco on the association of *H. pylori* genotypes with gastric histological profiles. We decided a priori to divide our studied population in two groups according to their age for several reasons. Firstly, *H. pylori* is contracted in childhood; and secondly, a loss of glands, resulting in multifocal atrophic gastritis can lead to the development of intestinal metaplasia and dysplasia and are linked to prolonged infection and consequently to patients’ age.

In our study, *H. pylori* was statistically significantly associated with CI, NA and GA but negatively with IM. These results (except the negative association with IM) confirm those obtained by Kalebi et al. reporting causal association of *H. pylori* with CI, NA and GA [21]. The natural history of gastric lesions begins with *H. pylori* infection leading to chronic gastritis that can progress to more severe lesions even in the absence of bacteria [22,23]. This hypothesis could explain, in part, the failure of *H. pylori* detection in severe lesions and also the negative association of this infection with IM. It’s also of interest to note that in our GC series, *H. pylori* has been detected with lower rates (57.5%) than those reported in Brazil (93%), USA (84.4%), neighboring North African country “Libya” (63.2%) and also than the mean rate reported in developed countries (61.5%) [1,24-26]. However, the number of cancer cases in this series is too low to confirm these results. The implication or causal association of virus with gastric carcinogenesis is plausible factor to explain the failure on *H. pylori* detection in some GC cases in our series. In effect, several studies have reported the implication of Epstein-Barr virus (EBV) in nearly 10% of gastric cancer cases [27-31] and Human papillomavirus (HPV) in other cases, particularly in India [32]. Therefore, the implication of those micro-organisms must be verified.

It has been well established that gastritis is the first lesion observed after *H. pylori* infection. This data can explain the statistically significant association of *H. pylori* with CI and NA (regardless of genotype) in the age group 1. Such association is lost in severe lesions (GA, IM and GC). Remarkably, *H. pylori* infection was significantly associated to those severe lesions in age group 2 (positively with GA and negatively with IM). It was hypothesized that patients exposed to long time infection with more virulent genotypes was at risk to develop atrophic gastritis and eventually gastric cancer via metaplasia. In spite of discrepancy on this subject, most studies report an association of *vacA* s1, *vacA* m1 and *cagA*+ genotypes with greater gastric epithelial damages and especially with GC [17,18,22,33-35]. This can be confirmed by the results obtained with the age group 2 in our study. A significant association between *vac A*m1 (p=0.04) and also *cag A* (p=0.04) was obtained in GC cases. However, *vacA*s1 was predominant (66.67%) in patients with GC, but with no significant association, which can be due to the low number of *H. pylori* positives cases in this group.. *Vac A*m1 and *cag A* genotypes were also predominant in SRCC which is a severe form of GC. Nevertheless, *vacA*s2m2 strains have been detected in 16% (n=4/25) of GC *H.pylori* positives cases. Those results let suppose that there are additional factors associated (virulence genes and environmental factors) to severe gastric epithelial damages and specially to GC. This hypothesis is strengthened by the low rate of GC comparatively to the very high rate of GA, which is considered as precancerous lesions [36]. 

In our series, high rate of glandular atrophy was observed when compared to that obtained in other African countries such as Kenya, Nigeria, Tunisia [21,37,38]. This raises a question about the factors implicated in this process. It’s known that *H. pylori* infection is generally contracted at early age. This acquisition in childhood associated [39] to the lower socioeconomic status, which prevents early diagnosis of infection, permits the persistence of this bacteria conducting to GA development. In our context, the rate of infection observed in adults aged <50 years (regardless of lesion), leads us to suppose that there is a high prevalence of infection in childhood. The persistence of this bacteria associated to environmental factors (or may be nutritional habits) leads to atrophy development. The environmental implication can be supported by the significant association of this lesion and also metaplasia with living area as observed in our studies (predominant in rural area). In other hand, and in spite of no significant association between *H.pylori* genotype and GA or IM in age group 1, the rate of less virulent genotypes in those lesions (s2 and m2 versus s1 and m1) was remarkable. This data can also support environmental implication or also, other genetic factors. To verify this hypothesis, a study on the prevalence of *H.pylori* infection in children, the implication of some nutritional and virulence factors are conducted in our region by our team. 

This is the first large sampling prospective study aiming to determine the histopathological profile of *H.pylori* in infected patients and its correlation to *vacA* genotypes and *cagA* status in Moroccan patients. It’s also the first study to explore these associations according to the age. In spite of some limitations (Only biopsies from the antrum were used for genotyping analysis in each patient, some discordance between PCR and histological exam results (that must be explained)), this study showed significant association between *cagA* ^+^ and NA in age group <50 years and with GC later in life (in age group > 50 years). Similarly, significant association was obtained between metaplasia and *H. pylori vacA s1* and *vacA* m1 genotypes in age group 2. This last genotype is also associated to GC. This study let suppose that the occurrence of GC in our region is relatively high with no significant association with *H.pylori*. However, the role of other etiological agents such as EBV and HPV and possibly environmental and dietetic factors must be explored. 
